# Investigation into Molecular Brain Aging in Senescence-Accelerated Mouse (SAM) Model Employing Whole Transcriptomic Analysis in Search of Potential Molecular Targets for Therapeutic Interventions

**DOI:** 10.3390/ijms241813867

**Published:** 2023-09-08

**Authors:** Michitaka Fujiwara, Farhana Ferdousi, Hiroko Isoda

**Affiliations:** 1 Graduate School of Environmental Science Program, University of Tsukuba, 1-1-1 Tennodai, Tsukuba 305-8577, Japan; 2 Open Innovation Laboratory for Food and Medicinal Resource Engineering, National Institute of Advanced Industrial Science and Technology (AIST), 1-1-1 Tennodai, Tsukuba 305-8572, Japan; 3 Faculty of Life and Environmental Sciences, University of Tsukuba, 1-1-1 Tennodai, Tsukuba 305-8572, Japan; 4 Alliance for Research on the Mediterranean and North Africa (ARENA), University of Tsukuba, 1-1-1 Tennodai, Tsukuba 305-8572, Japan

**Keywords:** SAMP8, cognitive aging, neurogenesis, synapse, neurometabolism, neuroinflammation

## Abstract

With the progression of an aging society, cognitive aging has emerged as a pressing concern necessitating attention. The senescence-accelerated mouse-prone 8 (SAMP8) model has proven instrumental in investigating the early stages of cognitive aging. Through an extensive examination of molecular changes in the brain cortex, utilizing integrated whole-genome transcriptomics, our principal aim was to uncover potential molecular targets with therapeutic applications and relevance to drug screening. Our investigation encompassed four distinct conditions, comparing the same strain at different time points (1 year vs. 16 weeks) and the same time point across different strains (SAMP8 vs. SAMR1), namely: physiological aging, accelerated aging, early events in accelerated aging, and late events in accelerated aging. Focusing on key functional alterations associated with aging in the brain, including neurogenesis, synapse dynamics, neurometabolism, and neuroinflammation, we identified candidate genes linked to these processes. Furthermore, employing protein–protein interaction (PPI) analysis, we identified pivotal hub genes involved in interactions within these functional domains. Additionally, gene-set perturbation analysis allowed us to uncover potential upstream genes or transcription factors that exhibited activation or inhibition across the four conditions. In summary, our comprehensive analysis of the SAMP8 mouse brain through whole-genome transcriptomics not only deepens our understanding of age-related changes but also lays the groundwork for a predictive model to facilitate drug screening for cognitive aging.

## 1. Introduction

In recent years, the steady rise in average life expectancy has brought about a decline in various physiological functions during old age, posing significant challenges to daily life and leading to the onset of diseases [1]. Among the organs affected, the brain stands out as one of the most vital and susceptible to age-related deterioration. Recent studies have elucidated multiple manifestations of brain dysfunction associated with aging, including diminished neuronal activity, loss of neuronal circuits, reduced synaptic plasticity, mitochondrial dysfunction, metabolic disturbances, and heightened neuroinflammatory responses [2,3,4,5]. These types of age-related brain dysfunctions have been closely linked to cognitive impairments collectively termed “cognitive aging” [6]. The advent of an aging society has fueled a surge of interest in cognitive aging, prompting extensive research at both genetic and cellular levels. While numerous biomarkers associated with cognitive aging have been identified, it remains challenging to establish a unified definition due to its multifactorial nature [7].

Simultaneously, extensive endeavors have been undertaken to explore drug discovery and development strategies, specifically focusing on cognitive aging [8,9]. However, despite substantial research efforts, pharmacological interventions have mainly demonstrated symptom alleviation without effectively targeting the underlying pathophysiological mechanisms of cognitive decline. Moreover, the clinical success of such interventions has been impeded by adverse side effects, limitations posed by the blood-brain barrier, and issues related to bioavailability. Therefore, in recent years, there has been a notable upsurge in interest surrounding the utilization of natural compounds as potential therapeutics. These compounds offer prolonged holistic effects and exhibit minimal toxicity, making them viable options for both preventive and therapeutic measures. Many of these bioactive compounds possess neurotrophic and neuroprotective properties, targeting essential biological pathways involved in cognition. While a significant number of promising bioactive compounds with favorable cognitive effects have been identified, a key challenge lies in the scarcity of studies employing a unified prediction model within this field, particularly considering the inherent multi-target potential of these compounds.

The senescence-accelerated mouse-prone 8 (SAMP8) mouse model, developed by Professor Takeda and colleagues at the Department of Pathology, Chest Disease Research Institution, Kyoto University, exhibits an early onset of cognitive aging characterized by impairments in learning and memory [10,11]. SAMP8 is one of the strains within the broader senescence-accelerated mouse (SAM) model, which comprises both SAMP (senescence-accelerated mouse-prone) and SAMR (senescence-accelerated mouse-resistant) strains. While SAMP strains undergo an accelerated aging process, SAMR1 strains experience a normal aging process [12]. Recent studies have revealed age-related morphological changes in SAMP8, including abnormal glial responses, increased phosphorylated tau levels, and early accumulation of amyloid in the hippocampus [13,14]. These characteristics are thought to contribute to cognitive dysfunction, making SAMP8 an excellent animal model for studying age-related cognitive decline.

Numerous valuable studies have shed light on the age-related alterations in the brain of the SAMP8 mouse model. Some investigations have focused on comparing SAMP8 with SAMR1 strains at the same time point [15,16], while others have examined the time-based comparisons within the same SAMP8 strain (e.g., 12 months vs. 6 months) [17]. However, there is a dearth of studies that integrate both the comparison of the same SAMP8 strain at different time points and the comparison of different strains (SAMP8 vs. SAMR1) at the same time point. Employing this integrated approach may provide a more comprehensive understanding of the age-related changes occurring in the brain of the SAMP8 mouse model.

In our current investigation, our primary objective entailed the establishment of a predictive model for cognitive aging through an exploration of the underlying mechanisms involved in age-related changes in the brain cortex of the SAMP8 mouse. Leveraging transcriptomics analysis, we conducted comparative analyses across two distinct dimensions, encompassing the comparison of the same strain at different time points (1 year vs. 16 weeks) to assess both physiologic and accelerated changes of aging and the comparison of different strains (SAMP8 vs. SAMR1) at the same time point to capture the evolving early and late events within the SAMP8 model. Our study aimed to provide a valuable foundation for a predictive model that may effectively elucidate the intricate processes associated with cognitive aging while simultaneously serving as a suitable tool for screening potential biomolecules targeting multifaceted aspects of cognitive aging.

## 2. Results

### 2.1. Characterization of Gene Expression Profiles in SAMR1 and SAMP8 Mice

We performed whole-genome RNA microarray analysis to investigate the changes of brain functions with aging at the molecular level in four conditions (physiological aging, accelerated aging, early events in accelerated aging, and late events in accelerated aging). Please refer to Table 1 for a summary of the comparisons performed.

We observed the differential expression of gene symbols in different conditions. Specifically, we identified 6230 gene symbols as differentially expressed in physiological aging, 4812 gene symbols in accelerated aging, 1759 gene symbols in early events in accelerated aging, and 2851 gene symbols in late events in accelerated aging. The top 10 up- and downregulated differentially expressed genes (DEGs) and related biological functions in all four conditions are given in Table 2 and Table 3.

**Figure 1:**
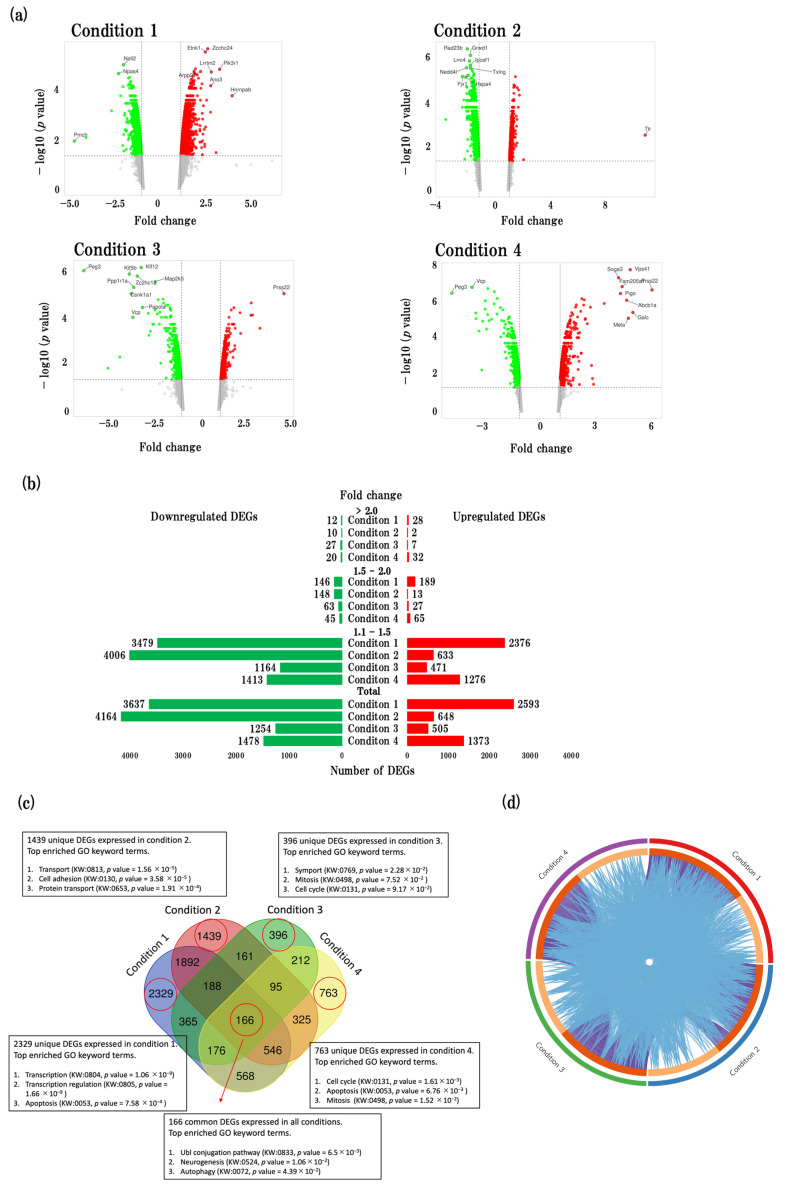
Characterization of gene expression profiles in SAMR1 and SAMP8 mice. (**a**) Volcano plots show DEGs in four conditions. The vertical axis (*y*-axis) corresponds to −log10 (*p* value) and the horizontal axis (*x*-axis) displays linear fold change. The red dots represent the upregulated genes; the green dots represent the downregulated genes. The top 10 DEGs with the biggest fold changes are shown in the figure. (**b**) Bar graphs showing the distribution of fold changes. The red bars represent the number of upregulated DEGs; the green bars represent the number of downregulated DEGs. (**c**) Venn diagram showing unique and common DEGs in four conditions. Significantly enriched gene ontology (GO) terms are listed for each set of DEGs. Enriched GO terms identified using David online tool. (**d**) Circos plot showing overlap genes and biological functions of each condition. Purple curves showing linking identical genes; blue curve shows linking identical biological functions. Circos plot was made by using Metascape tool (https://metascape.org/gp/index.html#/main/step1) (accessed on 3 July 2023).

The volcano plots provide the representation of the DEGs across four conditions, showcasing the top 10 most significantly expressed genes (Figure 1a). The upregulated DEGs are represented by red dots, while downregulated DEGs are represented by green dots. The distribution of fold changes in up- and downregulated genes is shown in the bar graph (Figure 1b).

The Venn diagram (Figure 1c) illustrates the distribution of unique and shared genes across the four conditions. We identified 2329, 1439, 396, and 763 unique DEGs in physiological aging, accelerated aging, early events in accelerated aging, and late events in accelerated aging, respectively. Additionally, we identified 166 DEGs that were common to all four conditions. Furthermore, we conducted an analysis to identify the top gene ontology (GO) keyword terms enriched by gene sets from the unique DEGs in each condition (transport, transcription, apoptosis, mitosis, cell cycle) as well as the common gene set across all four conditions (UbI conjugation pathway, neurogenesis, and autophagy) (Figure 1c).

Our findings indicate that a considerable number of DEGs and biological functions are shared among the four conditions (Figure 1d). Notably, functional overlaps were more prominent than overlaps in the specific DEGs identified in each condition.

### 2.2. Overview of Biological Events in SAMR1 and SAMP8 Mice

**Figure 2:**
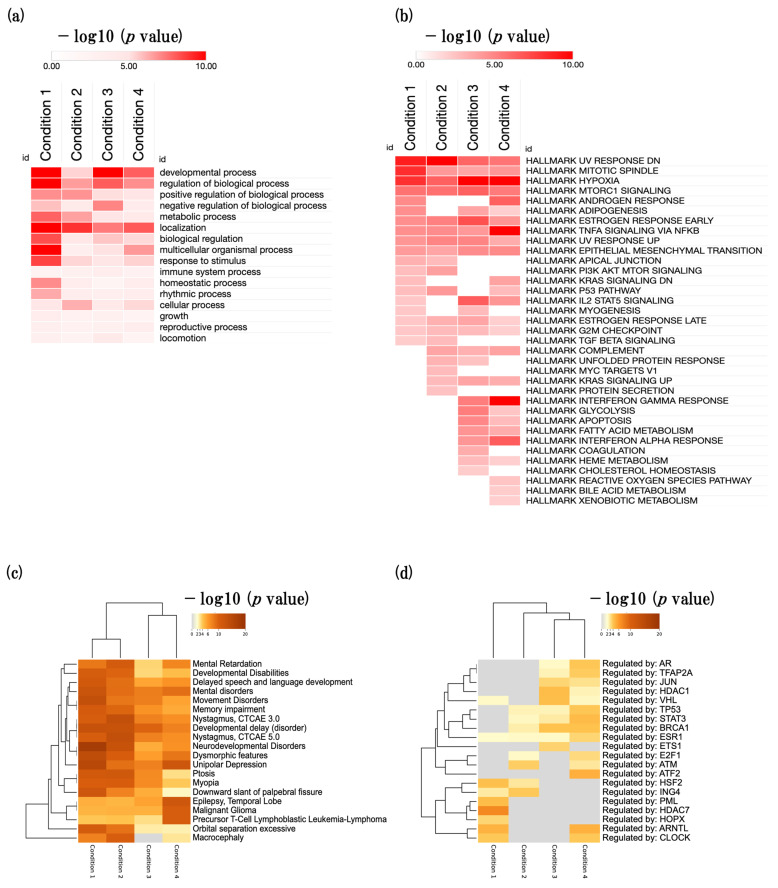
Overview of biological events in SAMR1 and SAMP8 mice. (**a**) Heatmap showing the parent GOBP terms in four conditions. (**b**) Heatmap showing the hallmark gene sets. (**c**) Heatmap showing disease enrichment analysis (DisGeNET database). (**d**) Heatmap showing TF enrichment analysis (TRRUST database). The color code represents the −log10 (*p* value). Enrichment analyses were conducted using the Metascape tool v3.5.20230101 (https://metascape.org/gp/index.html#/main/step1) (accessed on 8 May 2023). Heat map was generated using the Morpheus tool (https://software.broadinstitute.org/morpheus/) (accessed on 8 May 2023).

Next, we performed biological process gene ontology (GOBP) analysis in four conditions and identified significantly enriched parent GOBP terms. The developmental process (GO:0032502), regulation of biological process (GO:0050789), positive regulation of biological process (GO:0048518), negative regulation of biological process (GO:0048519), biological regulation (GO:0065007), metabolic process (GO:0008152), response to stimulus (GO:0050869), and immune system process (GO:0002376) were significantly enriched (Figure 2a).

Next, we performed an enrichment analysis of hallmark gene sets using MSigDB in four conditions. We identified many developmental-, metabolism-, and inflammation-related hallmark gene sets that were significantly enriched, such as mitotic spindle, epithelial mesenchymal transition, hypoxia, interferon gamma response, and adipogenesis (Figure 2b, Supplementary File S1).

Next, we performed disease-gene association enrichment analysis using the DisGeNET database in four conditions [18]. We found that cognitive-related diseases such as delayed speech and language development, mental disorders, and memory impairment were enriched (Figure 2c).

Finally, we performed a transcription factor (TF) enrichment analysis using the TRRUST (transcriptional regulatory relationships unraveled by sentence-based text mining) database in all four conditions (Figure 2d) [19]. We identified several significantly enriched TFs associated with specific biological processes. Notably, we identified the enrichment of TFs related to synapse regulation (VHL), inflammation (STAT3, HDAC7), histone modifications (HDAC1, HDAC7, ING4), and RNA polymerase activity (E2F1, HOPX). Following the GOBP parent term enrichment analysis, we proceeded to select the four most enriched BP terms. These included (1) developmental process, (2) regulation of biological process, (3) metabolic process, and (4) response to stimulus and immune system process. By focusing on these highly enriched terms, we gained valuable insights into the underlying biological mechanisms at play in all four conditions.

### 2.3. Effects of Aging on Neurogenesis

#### 2.3.1. Overview of the Neurodevelopment-Related GOBPs

**Figure 3:**
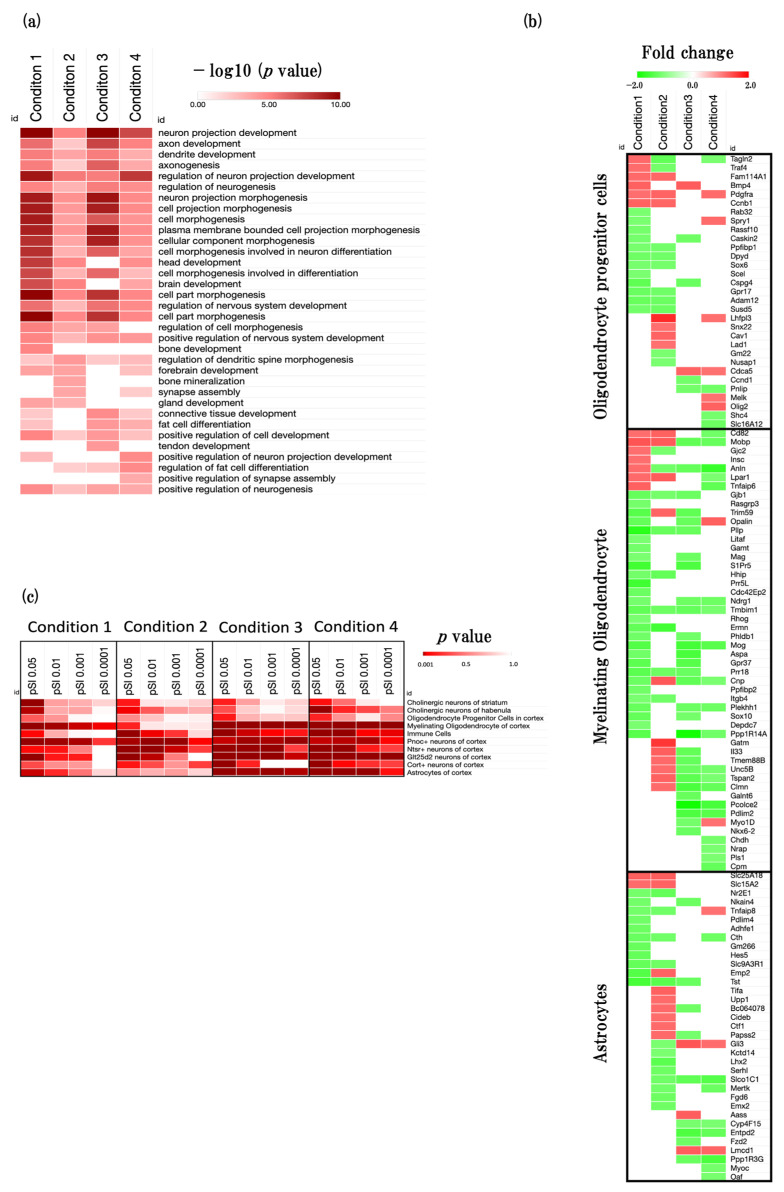
Effect of aging on neurodevelopment-associated biological function. (**a**) Heatmap showing GOBP terms related to developmental process (GO:0032502), presented as −log10 (*p* value). (**b**) Heatmap showing enrichment analysis for the specific cell types in the brain. Cell-type-specific enrichment analysis was conducted using the CSEA tool v 1.1 (http://genetics.wustl.edu/jdlab/csea-tool-2/) (accessed on 1 May 2023). (**c**) Heatmap showing the astrocytes and oligodendrocytes specific gene expression profiles, presented as fold change. The red color represents the upregulated DEGs, and the green color represents downregulated DEGs.

In order to ascertain the general pattern of developmental processes influenced by the aging process, we conducted an analysis using the web-based tool Metascape, focusing on developmental-process-related GOBPs (GO:0032502). We found numerous functional enrichments specific to neurogenesis, including activities such as neuron projection development (GO:0031175), axon development (GO:0061564), and dendrite development (GO:0016358) across all four conditions (Figure 3a, Supplementary File S2).

#### 2.3.2. Cell-Type Changes during the Aging Process

To identify more detailed trends of neurogenesis in four conditions, we performed cell-type-specific enrichment analysis using the CSEA web tool (Figure 3 and Figure S1). Moreover, we performed specific genes expression analysis of oligodendrocyte progenitor cells, myelinating oligodendrocytes, and astrocytes (Figure 3c, Supplementary File S3).

In physiological aging, we identified that the function of myelinating oligodendrocytes, and Pnoc+ neurons were significantly enriched. In addition, we identified that myelin-associated oligodendrocytic basic protein (*Mobp*), bone morphogenetic protein 4 (*Bmp4*), solute carrier family 25 (*Slc25A18*), and cyclin B1 (*Ccnb1*) were significantly upregulated, and plasma membrane proteolipid (*Pllp*), proline rich 5 like (*Prr5L*), sphingosine-1-phosphate receptor 5 (*S1Pr5*), and thiosulfate sulfurtransferase, mitochondrial (*Tst*) were significantly downregulated.

In accelerated aging, we identified that the function of neurons such as Pnoc+ neurons, Ntsr+ neurons, and Cort+ neurons was significantly enriched. In addition, we identified lipoma HMGIC fusion partner-like 3 (*Lhfpl3*), glycine amidinotransferase (*Gatm*), 2′,3′-cycle nucleotide 3′phosphodiesterase (*Cnp*), and *Mobp* were significantly upregulated, and ermin, ERM-like protein (*Ermn*), LIM homeobox protein 2 (*Lhx2*), transgelin 2 (*Tagln2*), and *Tst* were significantly downregulated.

In early events in accelerated aging, we identified that the function of myelinating oligodendrocytes, astrocytes, immune cells, Pnoc+ neurons, Ntsr+ neurons, and Glt25d2 neurons was significantly enriched. In addition, we identified that GLI-Kruppel family member GLI3 (*Gli3*), LIM and cysteine-rich domains 1 (*Lmcd1*), aminoadipate-semialdehyde synthase (*Aass*), and *Bmp4* were significantly upregulated, and procollagen C-endopeptidase enhancer 2 (*Pcolce2*), protein phosphatase 1, regulatory inhibitor subunit 14A (*Ppp1R14A*), myelin oligodendrocyte glycoprotein (*Mog*), and aspartoacylase (*Aspa*) were significantly downregulated.

In late events in accelerated aging, we identified that the function of myelinating oligodendrocytes, astrocytes, immune cells, Pnoc+ neurons, Ntsr+ neurons, Glt25d2 neurons, and Cort+ neurons were significantly enriched. In addition, we identified that oligodendrocytic myelin paranodal and inner loop protein (*Opalin*), oligodendrocyte transcription factor 2 (*Olig2*), sprout RTK signaling antagonist 1 (*Spry1*), and *Lmcd1* were significantly upregulated, and anillin, actin binding protein (*Anln*), protein phosphatase 1, regulatory subunit 3G (*Ppp1R3G*), and solute carrier organic anion transporter family, member 1c1 (*Slco1C1*), and *Pcolce2* were significantly downregulated.

### 2.4. Effects of Aging on Synapse

#### 2.4.1. Overview of the Synapse-Related GOBPs

**Figure 4:**
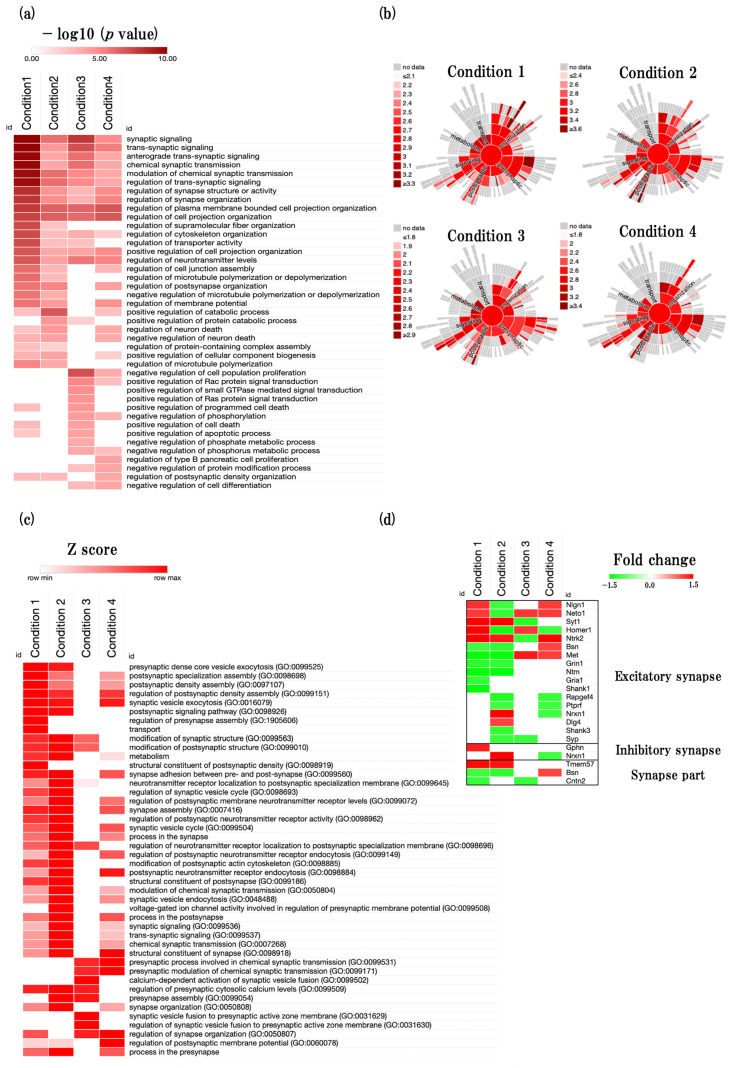
Effect of aging on synapse-associated biological functions. (**a**) Heatmap showing regulation of GOBP terms (GO:0050789, GO:0048518, GO:0048519), presented as −log10 (*p* value). (**b**) Sunburst graphs show the synapse-specific biological process gene ontology (GOBP) terms. Synapse-specific enrichment analysis was conducted using the SynGo tool (https://www.syngoportal.org/) (accessed on 2 May 2023). The red color represents the −log10 (*p* value). (**c**) Heatmap shows the synapse-specific GOBP terms, presented as z-score, identified from SynGo. (**d**) Heatmap showing synapse-related gene expression profiles, presented as fold change. The red color represents the upregulated DEGs, and the green color represents downregulated DEGs.

To identify the overall trend of the regulation of GOBP, we next performed the regulation of biological process (GO:0050789, GO:0048518, GO:0048519) analysis using the web-based tool Metascape. We identified that many synapses related GOBP terms such as synaptic signaling (GO:0099536), trans-synaptic signaling (GO:0099537), chemical synaptic transmission (GO:0007268), and regulation of synapse organization (GO:0050807) were significantly changed in all four conditions (Figure 4a, Supplementary File S2).

#### 2.4.2. Functional Changes during Aging Process of Synapse and Related Predicted Genes

We next performed the synapse-specific GOBP analysis using the web-based tool Synaptic Gene Ontologies (SynGo), which is a suitable tool for synapse-specific biological processes’ (BPs) and cellular components’ (CCs) analysis (Figure 4b,c, Supplementary File S4). In addition, we analyzed the expression of synapse-specific DEGs to identify the predicted genes changed with aging process (Figure 4d, Supplementary File S5).

In physiological aging, postsynaptic density assembly (GO:0097107), synaptic vesicle exocytosis (GO:0016079), and modification of synaptic structure (GO:0099563) were significantly enriched. In addition, we identified that synaptotagmin 1 (*Syt1*), neurotrophic tyrosine kinase, receptor, type 2 (*Ntrk2*), gephyrin (*Gphn*), and macoilin 1 (*Tmem57*) were significantly upregulated and bassoon (*Bsn*) and neurotrimin (*Ntm)* were significantly downregulated.

In accelerated aging, synaptic vesicle exocytosis (GO:0016079), modification of synaptic structure (GO:0099563), synapse assembly (GO:0007416), synaptic signaling (GO:0099536), and chemical synaptic transmission (GO:0007268) were significantly enriched. In addition, we identified that *Syt1*, *Ntrk2*, *Tmem57* were significantly upregulated and rap guanine nucleotide exchange factor (GEF) 4 (*Rapgef4*), SH3 and multiple ankyrin repeat domains 3 (*Shank3*), Synaptophysin (*Syp*), *Bsn*, and *Ntm* were significantly downregulated.

In early events in accelerated aging, the presynaptic process involved in chemical synaptic transmission (GO:0099531) and regulation of presynaptic cytosolic calcium levels (GO:0099509) were significantly enriched. In addition, we identified that neuropilin (NRP) and tolloid (TLL)-like 1 (*Neto1*), met proto-oncogene (*Met*) were significantly upregulated and *Syp* was significantly downregulated.

In late events in accelerated aging, postsynaptic signaling pathway (GO:0098926), postsynaptic neurotransmitter receptor endocytosis (GO:0098884), structural constituent of synapse (GO:0098918), and regulation of synapse organization (GO:0050807) were significantly enriched. In addition, we identified that *Neto1*, *Ntrk2*, *Bsn*, *Met* were significantly upregulated and *Rapgef4* was significantly downregulated.

### 2.5. Effects of Aging on Neuro-Metabolism

#### 2.5.1. Overview of the Neurometabolism Related GOBPs

**Figure 5:**
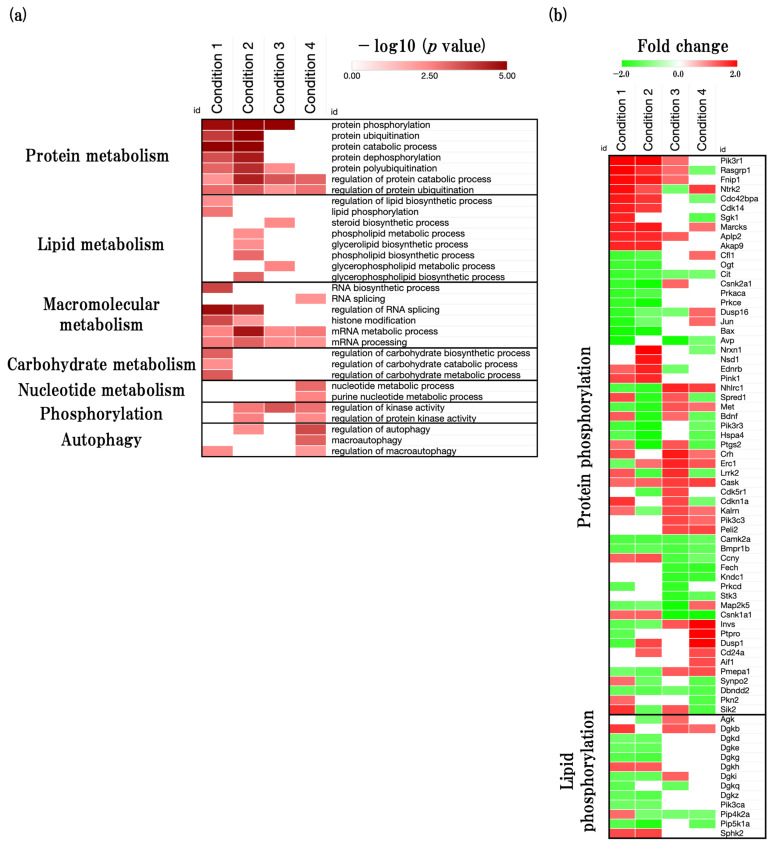
Effect of aging on neurometabolism-associated biological functions. (**a**) Heatmap shows GOBP terms related to metabolic process (GO:0008152), presented as −log10 (*p* value). (**b**) Heatmap shows protein-phosphorylation- and lipid-phosphorylation-related gene expression profiles, presented as fold change. The red color represents the upregulated DEGs, and the green color represents downregulated DEGs.

To identify the overall trends of metabolic processes, we next performed the metabolism-related GOBP (GO:0008152) analysis using the web-based tool Metascape. We identified that metabolism-related GOBPs such as protein metabolism, lipid metabolism, macromolecular metabolism, carbohydrate metabolism, nucleotide metabolism, phosphorylation, and autophagy were significantly enriched (Figure 5a, Supplementary File S2). We identified that protein-related BPs were significantly enriched in all conditions and especially in accelerated aging. Moreover, we identified that protein phosphorylation was significantly enriched in early events in accelerated aging, and this could be the cause of early onset of memory and learning deficit in SAMP8. We also found that the phospholipid metabolic process and glycerolipid biosynthetic process were significantly enriched in accelerated aging. Furthermore, we identified that autophagy was significantly enriched in late events in accelerated aging.

#### 2.5.2. Functional Changes during Aging Process of Neurometabolism and Related Predicted Gene

Next, we performed DEGs analysis of protein phosphorylation and lipid phosphorylation (Figure 5b, Supplementary File S6). In physiological aging, phosphoinositide-3-kinase regulatory subunit 1 (*Pik3r1*), RAS guanyl releasing protein 1 (*Rasgrp1*), folliculin interacting protein 1 (*Fnip1)*, and *Ntrk2* were significantly upregulated, and arginine vasopressin (*Avp*), BCL2-associated X protein (*Bax*), jun proto-oncogene (*Jun*), and dual specificity phosphatase 16 (*Dusp16*) were significantly downregulated. In accelerated aging, *Pik3r1*, neurexin 1 (*Nrxn1*), nuclear-receptor-binding SET-domain protein 1 (*Nsd1*), and myristoylated alanine rich protein kinase C substrate (*Marcks*) were significantly upregulated, and *Bax*, protein kinase C, epsilon (*Prkce*), prostaglandin-endoperoxide synthase 2 (*Ptgs2*), and casein kinase 2, and alpha 1 polypeptide (*Csnk2a1*) were significantly downregulated. In early events in accelerated aging, corticotropin-releasing hormone (*Crh*), ELKS/RAB6-interacting/CAST family member 1 (*Erc1*), NHL repeat containing 1 (*Nhlrc1*), and leucine-rich repeat kinase 2 (*Lrrk2*) were significantly upregulated, and *Avp*, casein kinase 1, alpha 1 (*Csnk1a1*), mitogen-activated protein kinase 5 (*Map2k5*), and protein kinase N1 (*Stk3*) were significantly downregulated. In late events in accelerated aging, inversin (*Invs*), protein tyrosine phosphatase receptor type O (*Ptpro*), dual specificity phosphatase 1 (*Dusp1*), and *Ntrk2* were significantly upregulated, and *Csnk1a1*, ferrochelatase (*Fech*), kinase non-catalytic C-lobe domain (KIND) containing 1 (*Kndc1*), and salt-inducible kinase 2 (*Sik2*) were significantly downregulated.

### 2.6. Effects of Aging on Neuroinflammation

#### 2.6.1. Overview of the Neuroinflammation-Related GOBPs

**Figure 6:**
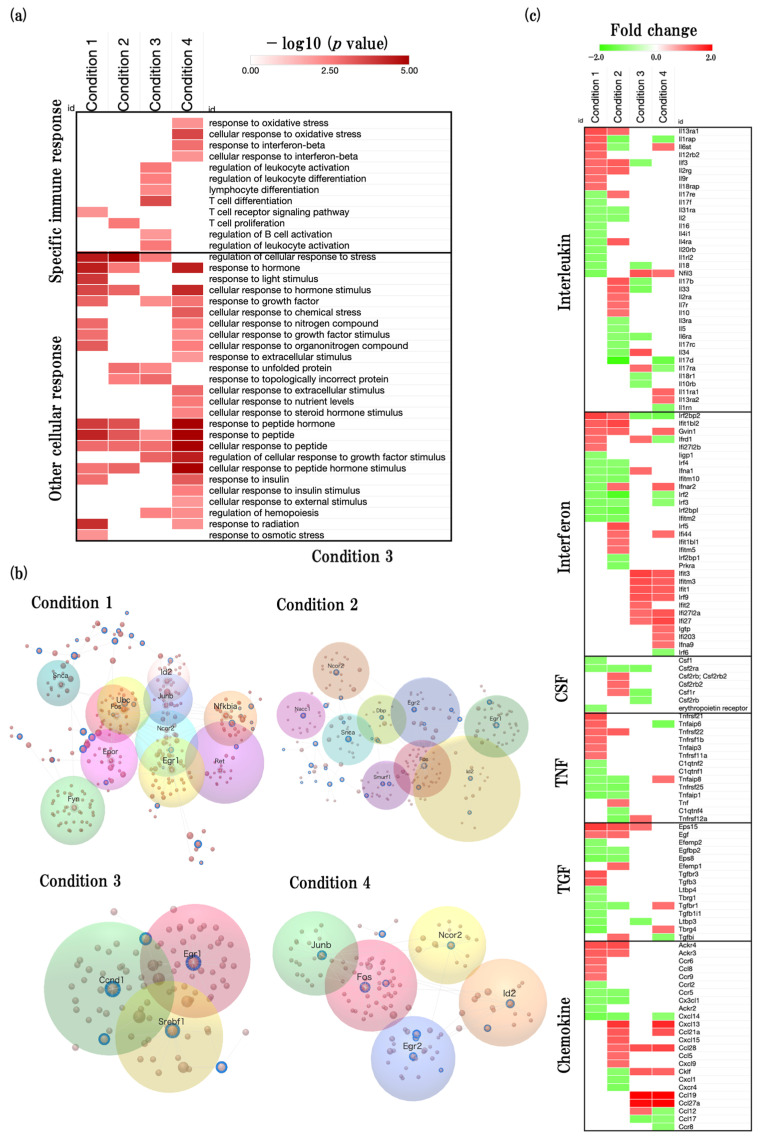
Effect of aging on neuroinflammation-associated biological functions. (**a**) Heatmap showing GOBP terms related to immune process (GO:0002376) and response to stimulus (GO:0050896), presented as −log10 (*p* value). (**b**) The inflammation-associated and functionally related gene modules are shown in the figure and key genes are shown at the center of each module. (**c**) Heatmap shows cytokine-related gene expression profiles (interleukin, interferon, colony stimulus factor (CNF), tumor necrosis factor (TNF), transforming growth factor (TGF), and chemokine) presented as fold change. The red color represents the upregulated DEGs, and the green color represents downregulated DEGs.

Next, we performed the response to stimulus and immune system processes related GOBP (GO:0050896, GO:0002376) analysis using the web-based tool Metascape. We found that transforming growth factor beta (TGF-β) and T-cell-related BPs were significantly enriched in physiological aging, T-cell- and interleukin-15- related BPs were significantly enriched in accelerated aging, interleukin-12-related BPs were significantly enriched in early events in accelerated aging, and interferon- and inflammatory-related BPs were significantly enriched in late events in accelerated aging (Figure 6a, Supplementary File S2). We also found that cellular response was significantly enriched in all conditions (Figure 6a).

#### 2.6.2. Functional Changes during Aging Process of Neuroinflammation and Related Predicted Genes

We next performed a gene network analysis of neuroinflammation-related genes changing with aging using the OmicsNet 2.0 web tool. We referred to the following paper to identify the neuroinflammation-related genes changing with aging [20].

We identified some key hub genes and modules of neuroinflammation (Figure 6b, Supplementary File S7). Moreover, we analyzed the expression of cytokine-specific DEGs to identify the predicted genes that changed with aging process (Figure 6c, Supplementary File S8).

In physiological aging, we found that the FBJ osteosarcoma oncogene (*Fos*) (degree = 53, expression = −1.44), followed by fyn proto-oncogene (*Fyn*) (degree = 45, expression = −1.31) are key predicted genes of inflammation. In addition, we also identified epidermal growth factor receptor pathway substrate 15 (*Eps15*), interferon regulatory factor 2 binding protein 2 (*Irf2bp2*), tumor necrosis factor receptor superfamily, member 21 (*Tnfrsf21*), and interleukin 13 receptor, alpha 1 (*Il13ra1*) were significantly upregulated, and chemokine (C-X-C motif) ligand 14 (*Cxcl14*), transforming growth factor beta regulated gene 4 (*Tbrg4*), epidermal growth factor receptor pathway substrate 8 (*Eps8*), and interferon-induced transmembrane protein 2 (*Ifitm2*) were significantly downregulated.

In accelerated aging, we found that *Fos* (degree = 53, expression = 1.54), followed by early growth response 1 (*Egr1*) (degree = 40, expression = −1.17) are the key predicted genes of inflammation. In addition to cytokine-specific DEGs, we identified that chemokine (C-X-C motif) ligand 13 (*Cxcl13*), interferon induced protein with tetratricopeptide repeats 1B like 2 (*Ifit1bl2*), atypical chemokine receptor 4 (*Ackr4*), and interferon regulatory factor 5 (Irf5) were significantly upregulated, and interleukin 17D (*Il17d*), interferon regulatory factor 2 (*Irf2*), interferon regulatory factor 2 binding protein-like (*Irf2bpl*), and interleukin 1 receptor accessory protein (*Il1rap*) were significantly downregulated.

In early events in accelerated aging, we found that *Egr1* (degree = 40, expression = 1.4), followed by cyclin D1 (*Ccnd1*) (degree = 28, expression = −1.18) are key predicted genes of inflammation. In addition to cytokine-specific DEGs, we identified that chemokine (C-C motif) ligand 19 (*Ccl19*), chemokine (C-C motif) ligand 27A (*Ccl27a*), interferon-induced protein with tetratricopeptide repeats 3 (*Ifit3*), and interferon-induced transmembrane protein 3 (*Ifitm3*) were significantly upregulated, and interleukin 33 (*Il33*), *Irf2bp2*, chemokine (C-C motif) ligand 17 (*Ccl17*), and colony stimulating factor 2 receptor, alpha, low-affinity (granulocyte-macrophage) (*Csf2ra*) were significantly downregulated.

In late events in accelerated aging, we found that *Fos* (degree = 53, expression = 2.25), followed by jun B proto-oncogene (*Junb*) (degree = 27, expression = 2.13) are the key predicted genes of inflammation. In addition to cytokine-specific DEGs, we identified that *Ccl19*, *Ccl27a*, *Cxcl13*, and chemokine (C-C motif) ligand 28 (*Ccl28*) were significantly upregulated, and *Il17d*, *Irf2bp2*, tumor necrosis factor alpha induced protein 6 (*Tnfaip6*), and *Il1rap* were significantly downregulated.

*Fos*, *Egr1*, *Snca*, *Ncor2*, and *Id2* were the common genes of physiological aging and accelerated aging. *Egr1* was the common genes of accelerated aging and early events in accelerated aging. *Fos*, *Egr2*, *Id2*, and *Ncor2* were the common genes of accelerated aging and late events in accelerated aging.

### 2.7. Protein–Protein Interaction Network Analysis

**Figure 7:**
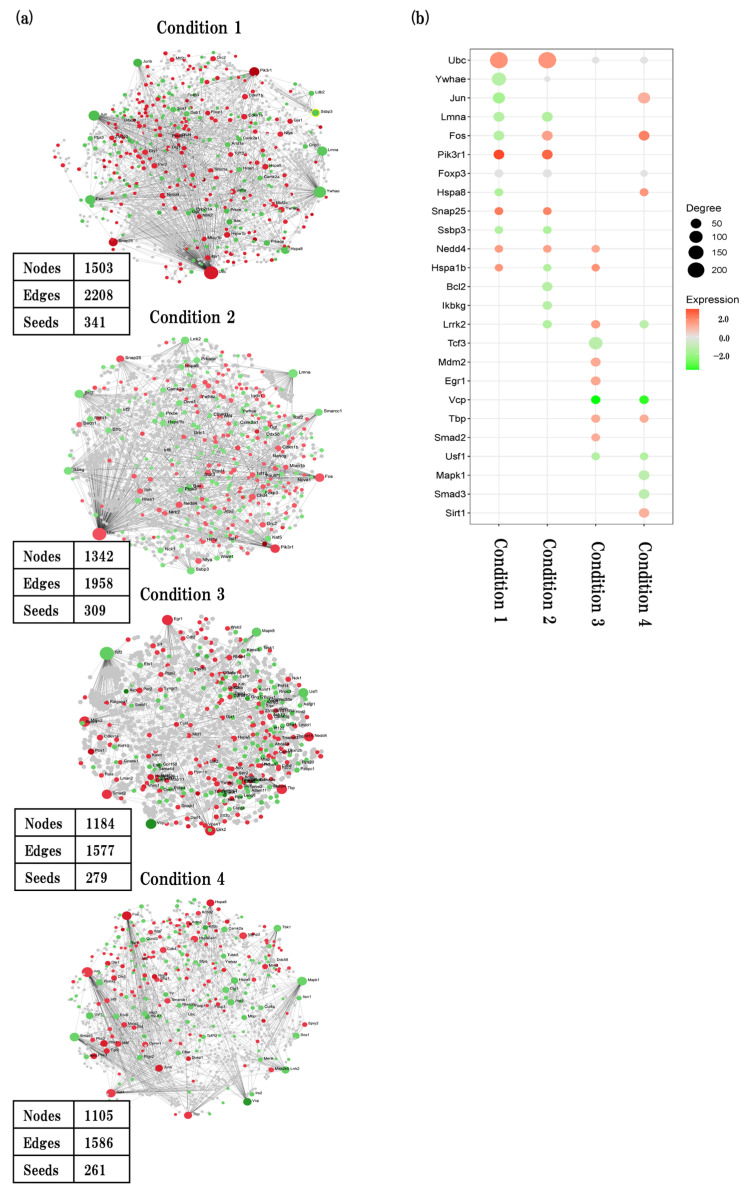
PPI interaction network analysis. (**a**) Generic first order PPI interaction network analysis based on IMEx interactome database showing key hub nodes in four conditions. (**b**) Bubble plots showing the corresponding top 20 hub genes (seeds) of 4 conditions. Red and green nodes represent up- and downregulated genes, respectively. Bubble size represents amount of degree.

We performed protein–protein interaction (PPI) network analysis to identify interacting genes and their associated biological functions (Figure 7). We built a first-order undirected PPI network using the NetworkAnalyst web-based tool. The node represents genes, and the edges represents gene–gene relation. The entire list of hub genes is provided in Supplementary File S9. We performed a genetic PPI analysis to identify the hub nodes, authenticated as the top nodes with highest degree in four conditions.

In physiological aging, a total of 341 seeds with 2208 edges were identified. ubiquitin C (*Ubc*) which is related to myelinating was the top hub node with the highest degrees (degree = 243, betweenness = 554264.28, expression = 1.9), followed by tyrosine 3-monooxgenase/tryptophan 5-monooxgenase activation protein, and epsilon polypeptide (*Ywhae*) (degree = 128, betweenness = 190779.12, expression = −1.38), which is related to synapse and axon formation.

In accelerated aging, a total of 309 seeds with 1958 edges were identified. *Ubc* was the top hub node with highest degrees (degree = 243, betweenness = 466012.45, expression = 1.82), followed by lamin A (*Lmna*) (degree = 54, betweenness = 63973.9, expression = −1.53) which is related to apoptotic signaling pathway.

In early events in accelerated aging, a total of 279 seeds with 1577 edges were identified. transcription factor 3 (*Tcf3)*, which is related to inflammation, was the top hub node with highest degrees (degree = 110, betweenness = 180435.67, expression = −1.29), followed by transformed mouse 3T3 cell doble minute 2 (*Mdm2*) (degree = 40, betweenness = 73186.98, expression = 1.37) which is blood vessel formation.

In late events in accelerated aging, a total of 261 seeds with 1586 edges were identified. jun proto-oncogene (*Jun*), which is related to neurons, was the top hub node with highest degrees (degree = 78, betweenness = 101022.93, expression = −1.19), followed by mitogen-activated protein kinase 1 (*Mapk1*) (degree = 53, betweenness = 72429.06, expression = 1.22) which is related to apoptosis.

### 2.8. Gene-Set Perturbation Analysis

**Figure 8:**
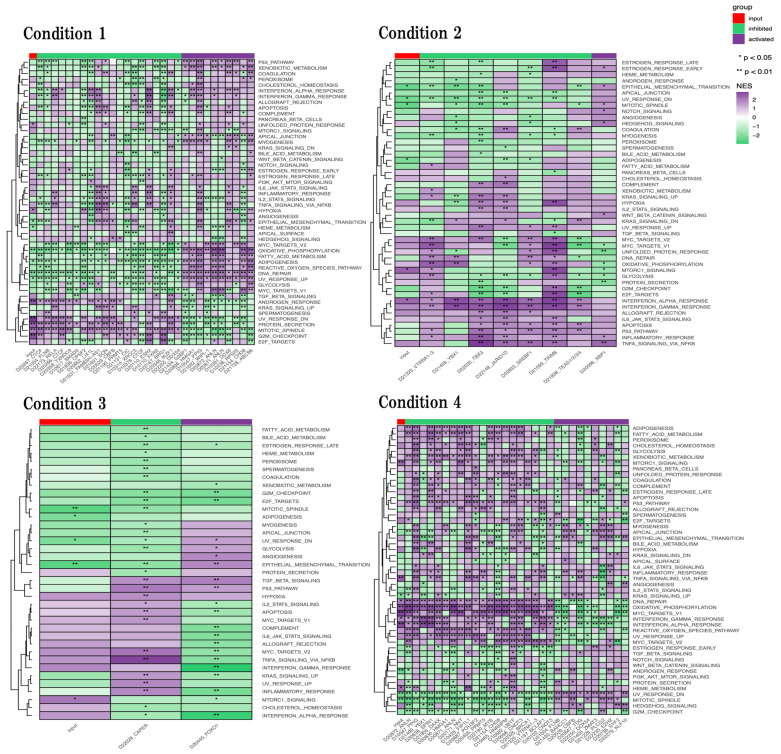
Genetic perturbation similarity analysis (GPSA) in four conditions. Purple and green codes represent activated and inhibited biological functions, respectively. The first column of each heatmap represents the activation/inhibition of the corresponding data set by the input gene list. * *p* < 0.05, and ** *p* < 0.01.

For a comprehensive understanding of biological functions, we next examined the activated-inhibited analysis of biological functions by using GPSA web tool (Figure 8).

In physiological aging, we identified that unfold protein response, hypoxia, protein secretion, mitotic spindle, and G2M checkpoint were significantly activated, and epithelial mesenchymal transition, heme metabolism, oxidative phosphorylation, adipogenesis, and DNA repair were significantly inhibited. We also found that inflammation-related functions such as Il6 JAK Stat3 signaling and inflammatory response were activated but not significantly.

In accelerated aging, we identified that Mtorc1 signaling, and Interferon alpha response were significantly activated, and epithelial mesenchymal transition, mitotic spindle, and adipogenesis were significantly inhibited. We also identified that inflammation-related functions such as Il2 STAT5 signaling, Il6 JAK Stat3 signaling, and TNFA signaling via NFKB were activated but not significantly.

In early events in accelerated aging, we identified that Mtorc1 signaling was significantly activated, and mitotic spindle, adipogenesis, and epithelial mesenchymal transition were significantly inhibited. We also identified that inflammation-related functions such as Il2 STAT5 signaling, Il6 JAK Stat3 signaling, TNFA signaling via NFKB, and interferon gamma response were activated but not significantly.

In late events in accelerated aging, we identified that Mtorc1 signaling, TNFA signaling via NFKB, DNA repair, oxidative phosphorylation, MYC targets V1, interferon gamma response, interferon alpha response, and heme metabolism were significantly activated, and upregulation of KRAS signaling, androgen response, and mitotic spindle were significantly inhibited. We also identified that G2M checkpoint was inhibited but not significantly.

## 3. Discussion

In our current investigation, we conducted a comprehensive examination of the brain cortex in SAMP8 mice, employing an integrated analysis of their genome and transcriptome. This analysis encompassed both temporal and functional aspects. The outcomes of our study propose some potential molecular targets to utilize SAMP8 mice as a predictive model for cognitive aging. SAMP8 mice may serve as a valuable resource for evaluating the functional characteristics of natural compounds, which may lead to the revelation of their diverse and manifold advantages.

The SAMP8 (senescence-accelerated mouse-prone 8) mouse model, developed by Dr. Takeda’s research team at Kyoto University, serves as an accelerated aging model, exhibiting the early onset of learning and memory deficits during the aging process [10,11,12]. Notably, cognitive decline in SAMP8 mice typically manifests between 2–4 months of age [21]. Moreover, SAMP8 mice display deficiencies in neurons and glial cells [22]. As a result, SAMP8 mice provide an excellent model for investigating cognitive aging, offering insights not only at the behavioral level but also at the pathological level. Emerging research has suggested the age-related alterations occurring in the brains of SAMP8 mice [15,16,17]. These investigations primarily concentrate on the temporal dynamics of SAMP8 or make comparisons between SAMP8 and SAMR1 mice. While these approaches have yielded significant advancements, the integration of both methodologies has been relatively scarce. By combining these approaches, there exists a promising opportunity to attain a comprehensive comprehension of the cognitive aging process in SAMP8 mice.

In the present study, we performed an enrichment analysis and found that developmental process, related to biological process, metabolic process, response to stimulus, and immune system process, was significantly enriched in all four conditions (Figure 2a). Moreover, we identified that hallmark gene sets such as mitotic spindle, hypoxia, adipogenesis, and epithelial mesenchymal transition, which are associated with the function of developmental process, metabolism, inflammation, were significantly enriched (Figure 2b). On the other hand, we found that many cognitive-decline-related diseases such as mental disorder, memory impairment, and developmental delay were significantly enriched (Figure 2c). Furthermore, we identified that synapse-related inflammation-related, histone-related, and RNA-polymerase-related TFs were significantly enriched (Figure 2d).

We performed developmental-process-specific BPs analysis and observed that many of these BPs exhibited a higher level of enrichment in physiological aging and early events of accelerated aging as opposed to the accelerated aging and late events in accelerated aging conditions (Figure 2a and Figure 3a). It is widely recognized that neurons, which directly influence memory and learning behaviors, tend to decrease with age in general. SAMP8 mice demonstrate neuronal loss starting at two months of age [22,23]. This observation aligns with the results of our research, as evidenced by the more significant alterations in the developmental process when we compared strains P8 and R1, but there were no substantial changes when we compared the two time points within the SAMP8 mice. Recent studies found that 16-week-old SAMP8 mice shows a decline in cognitive function [11,24]. Taken together, these studies and ours suggest a link between the early onset of functional changes of developmental process in the SAMP8 mouse brain and the early onset of cognitive decline in the SAMP8 mouse brain. Moreover, we found that astrocytes and myelinating oligodendrocytes were more significantly enriched in physiological aging, early events in accelerated aging, and late events in accelerated aging than accelerated aging (Figure 3b). These results also support the functional changes in the 16-week-old SAMP8 mouse brain. We also found that neuron-related functions were more significantly enriched in accelerated aging, early events in accelerated aging, and late events in accelerated aging than condition 1 (Figure 3b). Recent studies explain that astrocytes and oligodendrocytes play an important role for support neuron function, such as protecting and shaping neuron, synapse formation, and synaptic transmissions [25,26]. Taken together, early onset of astrocytes and oligodendrocyte functional changes may have an effect on neuronal functions. Thus, functional changes in astrocytes and oligodendrocytes in early time of aging process in SAMP8 may play an important factor of early onset of cognitive decline in SAMP8. Furthermore, we performed astrocyte- and oligodendrocyte-related DEGs expression analysis and identified the predicted gene sets (Figure 3c, Supplementary File S1). In this analysis, we identified some astrocytes and oligodendrocytes related genes such as *olig2* were significantly upregulated [27]. These results indicated that the reactive oligodendrocytes were significantly generated in the later stage of aging in SAMP8.

Next, we found that many synapse-related BPs were significantly changed in all conditions and noteworthy changes of synaptic-transmission-related BPs were identified in early events in accelerated aging (Figure 4a). Through a detailed synapse-specific enrichment analysis performed by SynGo online tool, we found that synaptic configuration-related and synaptic-transmission-related BPs such as synaptic vesicle exocytosis, modification of synaptic structure, metabolism, and synapse assembly were significantly enriched in physiological aging and accelerated aging. We also found that the synaptic-transmission-related BPs were more significantly changed in accelerated aging than physiological aging (Figure 4b,c). These results imply the dysfunction of synaptic-transmission-related functions were more remarkable in the SAMP8 mouse brain than the SAMR1 mouse brain. Moreover, we identified that synaptic-transmission-related BPs, such as those involved in the presynaptic process and in the chemical transmission and regulation of presynaptic cytosolic calcium levels, were significantly changed in the early events in accelerated aging. These results imply that changes in synaptic-transmission-related functions have already started in the 16-week-old SAMP8 mouse brain. In addition, we found that synaptic configuration-related and synaptic-transmission-related BPs were also significantly changed in late events in accelerated aging. These results suggest that synaptic-transmission-related function is reduced in the SAMP8 mouse brain prior to synaptic-configuration-related functions. Furthermore, we performed the excitatory and inhibitory synapse related DEGs expression analysis and identified the predicted gene sets (Figure 4d, Supplementary File S2).

Next, we also found that protein-metabolism-related BPs, such as protein phosphorylation, protein ubiquitination, and protein catabolic process, were significantly changed in all conditions especially accelerated aging has a marked tendency (Figure 5a). Protein metabolism plays an important role in maintain brain function through protein synthesis and degradation. In particular, the abnormal metabolism of tau protein is thought to be involved in more than 20 neurodegenerative disorders, including AD, and affects cognitive decline [28]. Interestingly, we also found that protein phosphorylation was significantly changed in early events in accelerated aging. The overproduction of phosphorylated tau protein, a type of phosphoprotein, seems to be associated with with tau aggregation and toxicity [29]. Moreover, because tau has been identified as a principal component of the neurofibrillary tangles (NFTs) changes of pathological features seen in the AD brain, the accumulation of phosphorylated and aggregated tau has been used for the staging of AD [30,31]. Given this suggestive evidence and our study, it is suggested that the early onset of functional changes in protein metabolism, especially protein phosphorylation, seems to be related to the early onset of cognitive decline in SAMP8.

We also found that the phospholipid metabolic process and glycerolipid biosynthetic process were significantly changed in accelerated aging. Recently, many studies have reported that phospholipids and glycerolipids seem to play a crucial role to maintain the synaptic transmission function [32,33,34]. Moreover, we found that the steroid biosynthesis process, which has been reported to play an important role in neurotransmitter release, was significantly enriched in early events in accelerated aging [35]. Given the early onset of functional changes of synaptic-transmission-related BPs in the SAMP8 mouse brain in our study and these studies, functional changes in synaptic-transmission-related BPs may be related to functional changes in phospholipid and glycerolipid metabolic processes, as well as the steroid synthesis metabolic process.

We also found that autophagy was significantly enriched in late events in accelerated aging. Autophagy plays an important role in degrading defective cells and misfold protein aggregates via lysosomes, and can also degrade the aberrant phosphorylation, aggregation, and proteolysis of tau protein, which seem to be associated with the development of AD [36]. In SAMP8 mice, it has been reported that the activity of autophagy decreases at the age of 12 months old. In contrast, SAMR1 mice do not show significant changes in autophagy at the age 12 months old [37]. These results provide greater confidence that the SAMP8 mouse brain experiences the dysfunction of autophagy in the late stages of aging.

Furthermore, we performed an analysis of protein phosphorylation and phospholipid metabolic processes related DEGs expression, and identified the predicted gene sets (Figure 5b, Supplementary File S3).

Next, we also found that many specific immune-response- and cellular-response-related BPs were significantly enriched (Figure 6a). In this study, we focus on specific immune-response-related functions and compare the age-associated inflammation functions which are recently studied to our study [20]. Through this comparison, we identified key hub-inflammation-related genes (Figure 6b, Supplementary File S4). Furthermore, we performed cytokine-related genes analysis and identified the predicted gene sets of cytokines (Figure 6c, Supplementary File S5).

Next, we confirmed key hub genes in PPI analysis to find the interacting genes with related biological functions (Figure 7a,b, Supplementary File S6).

Finally, we performed GPSA web tool to evaluate the active and inhibit behavior of biological functions (Figure 8).

In the line with neurogenesis and synapses, epithelial mesenchymal transition (EMT), which is the formation process of mesenchymal cells from epithelia was significantly inhibited in physiological aging, accelerated aging, and early events in accelerated aging [38]. Mesenchymal stem cells have been reported to differentiate into neuron and glial cells [39]. We also identified the unfold protein response (UPR), which is a cellular response to endoplasmic reticulum (ER) dysfunction, such as the aggregation of unfolded or misfolded proteins, was significantly activated in physiological aging and activated in accelerated aging (not significant) and inhibited (not significant) in late events in accelerated aging [40,41]. We also identified oxidative phosphorylation (OXPHOS), which is the production process of adenosine triphosphate(ATP) was significantly inhibited in physiological aging and significantly activated in accelerated aging and late events in accelerated aging. Recent studies found that the underproduction of ATP causes the dysfunction of mitochondria, while the overproduction of ATP produces reactive oxygen and causes DNA damage [42,43]. Moreover, we identified that DNA repair was significantly inhibited in physiological aging and significantly activated in late events in accelerated aging. Thus, the activation of OXPHOS in the SAMP8 mouse brain, especially in late aging, seem to induce DNA damage. In addition, we also identified that the mitotic spindle and G2M checkpoint, which are related to cell cycle, were significantly activated in physiological aging and inhibited in three other conditions, and significantly activated in physiological aging and inhibited in three other conditions, but not significantly, respectively [44,45]. Taken together, these results suggest that neuronal differentiation may seem to be dysfunctional in the 16-week-old SAMP8 mouse brain.

In the line with neurometabolism, we also found that adipogenesis was significantly inhibited in physiological aging, accelerated aging, and early events in accelerated aging. These results implied adipogenesis was already inhibited in the 16-week-old SAMP8 mouse brain. Adipogenesis is the process by which pre-adipocytes develop into mature adipocytes, and the over-production of adipocytes may induce neuronal dysfunction [46]. In our study, we did not observe the overproduction of adipocytes in the SAMP8 mouse brain. Thus, the SAMP8 mouse brain might not be a suitable model for the analysis of adipogenesis.

In the line with neuroinflammation, we also found that mTOR signaling was significantly activated in accelerated aging, early events in accelerated aging, and late events in accelerated aging. mTOR signaling plays an important role as a central regulator of cell metabolism, growth, proliferation, and survival [47,48]. In addition, mTOR signaling is involved in the regulation of autophagy, and the activation of mTOR signaling inhibits autophagy activity [49]. These studies imply that autophagy activity was inhibited in the SAMP8 mouse brain. This trend is similar to our results on the functional changes of autophagy in the late-stage SAMP8 mouse brain. We also found that hypoxia, which seems to induce inflammation, was significantly activated in physiological aging and was activated but not significantly in accelerated aging, early events in accelerated aging, and late events in accelerated aging [50]. These results implied that the hypoxia was already activated in the 16-week-old SAMP8 mouse brain and induced inflammation in early aging in the SAMP8 mouse brain. Inflammation-related functions such as interferon alpha response, interferon gamma response, Il6 JAK STAT3 signaling, and IL2 STAT5 signaling were activated in accelerated aging and early events in accelerated aging. Many studies confirm that inflammation-related functions were activated during the aging process [51]. These results were more supportive of advanced inflammation in the early aging process in the SAMP8 mouse brain.

## 4. Materials and Methods

### 4.1. Animals

In our in vivo experiments, we utilized male SAMP8 and SAMR1 mice at two different age groups: 16 weeks (*n* = 2) and 1 year (*n* = 2) (Japan SLC, Shizuoka, Japan). The mice were individually housed under carefully controlled conditions, including a temperature range of 21–23 °C and a light–dark cycle of 12 h each, while having unrestricted access to water and food.

### 4.2. RNA Extraction and Quantification

The mice were humanely euthanized by cervical dislocation, and their whole brains were carefully extracted. From each group, comprising 1-year-old and 16-week-old SAMR1 and SAMP8 mice, cerebral cortices were carefully dissected on ice for further analysis. The RNA samples were extracted using the Isogen kit (Nippon Gene Co. Ltd., Tokyo, Japan). Then, RNA quantity and quality were determined using the NanoDrop 2000 spectrophotometer (ThermoScientific, Waltham, MA, USA).

### 4.3. Microarray Experiment

Firstly, 100 ng of total RNA was utilized to synthesize double-stranded cDNA, employing the GeneAtlas 3′ IVT Express Kit (Affymetrix Inc., Santa Clara, CA, USA). Subsequently, biotin-labeled amplified RNA (aRNA) was synthesized via in vitro transcription, employing the GeneChip 3′ IVT Express Kit (Affymetrix Inc., Santa Clara, CA, USA). Following purification, a total of 9.4 mg of aRNA was fragmented using the GeneAtlas 3′ IVT Express Kit. The fragmented aRNA was then subjected to a 16 h hybridization at 45 °C, utilizing the GeneChip MG-430 PM microarray chip (Affymetrix Inc., Santa Clara, CA, USA). After hybridization, the microarray chip was subjected to a series of washes and staining in the Gene Atlas Fluidics Station 400 (Affymetrix Inc., Santa Clara, CA, USA). Finally, the resulting image was scanned using the GeneAtlas Imaging Station (Affymetrix Inc., Santa Clara, CA, USA).

### 4.4. Data Processing

The raw image data were normalized following the robust multichip average (RMA) algorithm using the Expression Console Software (Affymetrix, Japan, URL: http://www.affymetrix.com) (accessed on 6 December 2022). Subsequent gene expression analysis was carried out using freely available software Transcriptome Analysis Console (TAC) version 4 (Thermofisher Inc., Tokyo, Japan). We considered differentially expressed genes (DEGs) as *p*-value < 0.05, fold change > 1.1.

### 4.5. Date Analysis

In all analyses, we adapt top 1000 DEGs (Combined both 500 up- and downregulated DEGs). To investigate the changes in biological events in the aging brain, we established four distinct conditions for comparison. Firstly, we compared the molecular changes in brain cortices of SAMR1 mice, representing normal aging, between the ages of 1 year and 16 weeks. This comparison enabled us to examine the functional changes associated with the natural physiological aging process. Secondly, we compared the function changes in the brain cortices of SAMP8 mice, characterized by accelerated aging, between the ages of 1 year and 16 weeks. This allowed us to assess the functional changes specific to accelerated aging. Next, we compared SAMP8 and SAMR1 mice at two time points, 16 weeks and 1 year, to capture the trajectory of early and late events within the SAMP8 model. Please refer to Table 1 for a summary of the comparisons performed.

The volcano plots were generated using the VolcaNoseR web tool [52]. Heatmaps were created using the Broad Insititute’s Morphelus online tool (https://software.broadinstitute.org/morpheus/) (accessed on 8 May 2023).

Gene ontology (GO) enrichment analyses were performed using the web-based tools Metascape v3.5.20230101 (http://metascape.org) (accessed on 8 May 2023) [53] and David version 2021 (Database for Annotation, Visualization, and Integrated Discovery; URL: https://david.ncifcrf.gov/home.jsp) (accessed on 8 May 2023) [54].

Cell-type-specific expression analysis (CSEA) version1.1 tool was employed to identify potential cell populations that are likely to experience disruptions across our four analysis conditions. This tool utilizes a transcriptomic profiling dataset derived from mice to identify specific sets of transcripts that are expressed in distinct cell types. By employing Fisher’s exact test with Benjamini–Hochberg correction, CSEA identifies candidate gene lists that overlap with sets of transcripts enriched in specific cell types or regions (URL: http://genetics.wustl.edu/jdlab/csea-tool-2/) (accessed on 1 May 2023) [55].

Synaptic gene ontologies (SynGo) version1.1 web-based tool (URL: https://www.syngoportal.org/) (accessed on 2 May 2023) was used for synapse-specific analysis [56]. OmicsNet 2.0 web-based tool (URL: https://www.omicsnet.ca/) (accessed on 11 June 2023) was used for inflammation-specific analysis [57]. PPI networks were built using the NetworkAnalyst web-based tool version 3.0 (https://www.networkanalyst.ca/) (accessed on 14 June 2023) [58].

Genetic perturbation similarity analysis (GPSA) version 1.1.7 web-based tool was used to identify the upstream modulators and their activation/inhibition status across four different conditions (http://guotosky.vip:13838/GPSA/) (accessed on 26 April 2023) [59].

Gene–biological function relationships were analyzed using the web-based tool of Mouse genome Informatics and BioGPS (https://www.informatics.jax.org/, http://biogps.org/#goto=welcome) (accessed on 25 June 2023).

## 5. Conclusions

In summary, our study represents the first known report that employs a comprehensive approach, integrating whole genome transcriptomic analyses, to investigate cognitive aging in the SAMP8 mouse brain with respect to both temporal and functional factors. Through our analysis, we have identified numerous target functions and genes that are closely linked to the aging process in the SAMP8 mouse brain. Consequently, our findings hold significant potential for advancing drug discovery and development strategies targeted at mitigating cognitive aging-related conditions.

## Figures and Tables

**Table 1:** SAMP8 and SAMR1 comparison condition.

Condition	Comparison	Explanation
Condition 1	SAMR1: 1 year old vs. 16 weeks old	physiological aging
Condition 2	SAMP8: 1 year old vs. 16 weeks old	accelerated aging
Condition 3	16 weeks old: SAMP8 vs. SAMR1	early events in an accelerated aging
Condition 4	1 year old: SAMP8 vs. SAMR1	late events in an accelerated aging

**Table 2:** List of top 10 significantly upregulated genes and their functions in four conditions.

Gene Symbol	Description	Fold Change	*p* Value	Biological Functions
Condition 1				
*Hnrnpab*	heterogeneous nuclear ribonucleoprotein A/B	3.98	2.0 × 10^−4^	DNA binding, RNA binding, protein binding, nucleus, epithelial to mesenchymal transition, and dendrite
*Pik3r1*	phosphatidylinositol 3-kinase, regulatory subunit, polypeptide 1 (p85 alpha)	3.28	1.74 × 10^−5^	kinase activator activity, protein binding, ATPase binding, insulin receptor signaling pathway, and B cell differentiation
*Lrrtm2*	leucine rich repeat transmembrane neuronal 2	2.81	2.24 × 10^−5^	synapse organization, excitatory synapse, glutamatergic synapse, GABA-ergic synapse
*Ano3*	anoctamin 3	2.78	7.93 × 10^−5^	chloride channel activity, lipid transport, and calcium-activated phosphatidylcholine scrambling
*Homer1*	homer homolog 1 (Drosophila)	2.77	9.0 × 10^−4^	protein binding, molecular adaptor activity, dendrite, structural constituent of postsynapse, postsynaptic density, and excitatory synapse
*Zcchc24*	zinc finger, CCHC domain containing 24	2.61	2.64 × 10^−6^	nucleic acid binding
*Rasgrp1*	RAS guanyl releasing protein 1	2.55	3.0 × 10^−4^	calcium ion binding, inflammatory response to antigenic stimulus, natural killer cell activation, cell differentiation, T cell activation, and B cell activation
*Klhl24*	kelch-like 24	2.55	3.5 × 10^−3^	protein ubiquitination, regulation of kainate selective glutamate receptor activity, cytoplasm, andcell projection
*Etnk1*	ethanolamine kinase 1	2.48	3.54 × 10^−6^	nucleotide binding, ethanolamine kinase activity, ATP binding, kinase activity, lipid metabolic process, biosynthetic process, and phosphorylation
*Map2*	microtubule-associated protein 2	2.44	1.69 × 10^−2^	Protein binding, microtubule binding, axon genesis, dendrite development, and postsynapse
Condition 2				
*Ttr*	transthyretin	10.94	3.5 × 10^−3^	protein binding, purine nucleobase metabolic process, extracellular region, and extracellular space
*Apold1*	apolipoprotein L domain containing 1	1.82	5.72 × 10^−5^	lipid binding, angiogenesis, and regulation of endothelial cell differentiation
*Cyp2c29*	cytochrome P450, family 2, subfamily c, polypeptide 29	1.76	3.36 × 10^−5^	steroid hydroxylase activity, long-chain fatty acid omega-1 hydroxylase activity, lipid metabolic process, cytoplasm, and endoplasmic reticulum
*Lncpint*	long non-protein coding RNA, Trp53 induced transcript	1.74	2.0 × 10^−4^	skeletal system development, tissue development, and adipose tissue development
*Rbm12b1*	RNA binding motif protein 12 B1	1.66	2.66 × 10^−5^	nucleic acid binding, RNA binding, regulation of RNA splicing, and nucleoplasm
*Itih3*	inter-alpha trypsin inhibitor, heavy chain 3	1.62	1.4 × 10^−3^	peptidase inhibitor activity, extracellular region, and collagen-containing extracellular matrix
*Gatm*	glycine amidinotransferase (L-arginine:glycine amidinotransferase)	1.6	5.0 × 10^−4^	amidinotransferase activity, creatine biosynthetic process, learning or memory, and mitochondrion
*Btg2*	B cell translocation gene 2, anti-proliferative	1.59	1.06 × 10^−2^	protein binding, DNA damage response, neuron differentiation, and apoptotic process
*Defa15*	Defensin, alpha,15	1.53	2.2 × 10^−3^	defense response and extracellular region
*Fos*	FBJ osteosarcoma oncogene	1.52	4.4 × 10^−3^	DNA binding and cellular response to reactive oxygen species
Condition 3				
*Prss22*	protease, serine 22	4.69	9.43 × 10^−6^	peptidase activity and extracellular space
*Dctn3*	dynactin 3	2.91	7.96 × 10^−5^	cell cycle, cell division, kinetochore, nucleolus, and cytosol
*Adat2*	adenosine deaminase, tRNA-specific 2	2.84	2.39 × 10^−5^	catalytic activity and tRNA processing
*Vps52*	vacuolar protein sorting 52 (yeast)	2.82	8.21 × 10^−5^	protein targeting, Golgi to vacuole transport, protein transport, endosome
*Rpp25l*	ribonuclease P/MRP 25 subunit-like	1.91	1.7 × 10^−3^	nucleic acid binding, RNA binding, and tRNA 5′-leader removal
*Hspa1b*	heat shock protein 1B	1.91	2.4 × 10^−3^	protease binding, ATP binding, protein folding, nucleus, mitochondrion, and cell body
*Abcb1a*	ATP-binding cassette, sub-family B (MDR/TAP), member 1A	1.82	2.0 × 10^−4^	nucleotide binding, ATP binding, and G2/M transition of mitotic cell cycle
*Klf10*	Kruppel-like factor 10	1.81	2.9 × 10^−3^	DNA binding, protein binding, and rhythmic process
*Crh*	corticotropin releasing hormone	1.81	5.5 × 10^−3^	positive regulation of protein phosphorylation, inflammatory response, and learning and memory
*Vip*	vasoactive intestinal polypeptide	1.76	2.0 × 10^−4^	signaling receptor binding and learning and memory
Condition 4				
*Prss22*	protease, serine 22	6.1	2.59 × 10^−7^	peptidase activity
*Galc*	galactosylceramidase	5.06	4.3 × 10^−6^	galactosylceramidase activity, lipid metabolic process, myelination, and mitochondrion
*Vps41*	vacuolar protein sorting 41 (yeast)	4.91	2.08 × 10^−8^	protein binding, microtubule binding, autophagy, protein transport, cytoplasm, and endosome
*Abcb1a*	ATP-binding cassette, sub-family B (MDR/TAP), member 1A	4.72	9.37 × 10^−7^	nucleotide binding, ATP binding, transmembrane transporter activity, G2/M transition of mitotic cell cycle, phospholipid translocation, and cytoplasm
*Pigo*	phosphatidylinositol glycan anchor biosynthesis, class O	4.38	4.08 × 10^−7^	protein binding, transferase activity, endoplasmic reticulum, and membrane
*Soga3*	SOGA family member 3	4.28	5.69 × 10^−8^	regulation of autophagy, extracellular space, and membrane
*Cdhr1*	cadherin-related family member 1	2.92	3.37 × 10^−2^	calcium ion binding, protein binding, cell adhesion, cell-cell adhesion, and membrane
*Npas4*	neuronal PAS domain protein 4	2.86	1.63 × 10^−2^	DNA binding, protein binding, learning, cell differentiation, and inhibitory synapse assembly
*Btg2*	B cell translocation gene 2, anti-proliferative	2.81	3.2 × 10^−3^	protein binding, neuron differentiation, and apoptotic process
*Dctn3*	dynactin 3	2.75	2.24 × 10^−6^	cell cycle, cell division, kinetochore, and cytosol

**Table 3:** List of top 10 significantly downregulated genes and their functions in four conditions.

Gene Symbol	Description	Fold Change	*p* Value	Biological Functions
Condition 1				
*Pmch*	pro-melanin-concentrating hormone	−4.88	1.29 × 10^−2^	signaling receptor binding, positive regulation of cytosolic calcium ion concentration, chemical synaptic transmission, and dopaminergic
*Avp*	arginine vasopressin	−4.23	9.3 × 10^−3^	protein kinase activity, positive regulation of cytosolic calcium ion concentration, locomotory behavior, negative regulation of apoptotic process, ERK1 and ERK2 cascade, and dendrite
*Atp1a3*	ATPase, Na^+^/K^+^ transporting, alpha 3 polypeptide	−2.79	8.0 × 10^−4^	nucleotide binding, amyloid-beta binding, transporter activity, potassium ion transport, memory, intracellular potassium ion homeostasis, myelin sheath, and synapse
*Npas4*	neuronal PAS domain protein 4	−2.4	2.58 × 10^−5^	DNA binding, protein binding, cell differentiation, regulation of synaptic transmission, GABAergic, excitatory postsynaptic potential, inhibitory postsynaptic potential, and nucleus
*Fjx1*	four jointed box 1 (Drosophila)	−2.28	2.0 × 10^−4^	cell–cell signaling, extracellular region, and extracellular space
*Slc17a6*	solute carrier family 17 (sodium-dependent inorganic phosphate cotransporter), member 6	−2.16	1.93 × 10^−2^	chloride channel activity, neurotransmitter transmembrane transporter activity, neurotransmitter transport, synaptic transmission, glutamatergic, and synaptic vesicle
*Bax*	BCL2-associated X protein	−2.15	8.65 × 10^−5^	protein binding, channel activity, leukocyte homeostasis, T cell homeostatic proliferation, B cell homeostasis, myeloid cell homeostasis, apoptotic process, and mitochondrial fusion
*Nell2*	NEL-like 2	−2.12	1.15 × 10^−5^	protein kinase C binding, neuron cellular homeostasis, extracellular region, and dendrite
*Junb*	jun B proto-oncogene	−2.11	7.0 × 10^−3^	DNA binding, transcription factor binding, osteoblast differentiation, cell differentiation, regulation of cell cycle, nucleus, and nucleoplasm
*Cabp7*	calcium binding protein 7	−2.1	3.2 × 10^−2^	calcium ion binding and Golgi apparatus
Condition 2				
*Homer1*	homer homolog 1 (Drosophila)	−3.52	7.0 × 10^−4^	signaling receptor binding, protein binding, molecular adaptor activity, structural constituent of postsynapse, regulation of postsynaptic neurotransmitter receptor activity, regulation of dendritic spine maintenance, axon, dendrite, neuron projection, excitatory synapse, and glutamatergic synapse
*Fjx1*	four jointed box 1 (Drosophila)	−2.31	8.46 × 10^−6^	cell–cell signaling, extracellular region, and extracellular space
*Bax*	BCL2-associated X protein	−2.31	2.0 × 10^−4^	protein binding, lipid binding, neuron migration, leukocyte homeostasis, T cell homeostatic proliferation, B cell homeostasis, myeloid cell homeostasis, and apoptotic process
*Slc23a2*	solute carrier family 23 (nucleobase transporters), member 2	−2.2	9.38 × 10^−5^	transporter activity, transmembrane transporter activity, brain development, and cytoplasm
*Atp1a3*	ATPase, Na^+^/K^+^ transporting, alpha 3 polypeptide	−2.09	2.0 × 10^−4^	nucleotide binding, amyloid-beta binding, transporter activity, ATP binding, protein-folding chaperone binding, potassium ion transport, memory, cellular response to amyloid-beta, nucleus, cytoplasm, Golgi apparatus, axon, myelin sheath, synapse, and postsynapse
*Hspa4*	heat shock protein 4	−2.08	9.06 × 10^−6^	nucleotide binding, protein binding, ATP binding, negative regulation of protein phosphorylation, protein folding, neuron apoptotic process, regulation of microglial cell activation, nucleus, cytoplasm, lipid droplet, cytosol, and extracellular exosome
*Csnk2a1*	casein kinase 2, alpha 1 polypeptide	−2.07	2.0 × 10^−4^	nucleotide binding, protein kinase activity, protein binding, ATP binding, kinase activity, protein phosphorylation, apoptotic process, cell cycle, Wnt signaling pathway, phosphorylation, rhythmic process, regulation of cell cycle, chromatin, nucleus, and cytosol
*Rab4a*	RAB4A, member RAS oncogene family	−2.01	3.0 × 10^−4^	nucleotide binding, ATPase activator activity, GTPase activity, cytoplasm, endosome, membrane, synaptic vesicle membrane, recycling endosome, and extracellular exosome
*Nedd4l*	neural precursor cell expressed, developmentally down-regulated gene 4-like	−2.01	3.43 × 10^−6^	ubiquitin-protein transferase activity, protein binding, regulation of membrane depolarization, protein ubiquitination, cell differentiation, cytoplasm, endosome, Golgi apparatus, and plasma membrane
*Swi5*	SWI5 recombination repair homolog (yeast)	−2	2.53 × 10^−5^	Protein binding, DNA repair, and DNA damage response
Condition 3				
*Peg3*	paternally expressed 3	−6.63	9.43 × 10^−7^	DNA binding, regulation of transcription by RNA polymerase II, and apoptotic process,
*Pmch*	pro-melanin-concentrating hormone	−5.26	1.61 × 10^−2^	signaling receptor binding, positive regulation of cytosolic calcium ion concentration, chemical synaptic transmission, regulation of neuronal synaptic plasticity, extracellular region, and extracellular space
*Avp*	arginine vasopressin	−4.59	5.3 × 10^−3^	protein kinase activity, neuropeptide hormone activity, signal transduction, locomotory behavior, positive regulation of cell growth, apoptotic process, ERK1 and ERK2 cascade, dendrite, and neuronal dense core vesicle
*Kif5b*	kinesin family member 5B	−4.05	1.34 × 10^−6^	nucleotide binding, protein binding, ATP binding, microtubule binding, mitochondrial transport, axon guidance, synaptic vesicle transport, axonal growth cone, and endocytic vesicle
*Csnk1a1*	casein kinase 1, alpha 1	−3.94	9.54 × 10^−6^	nucleotide binding, protein kinase activity, ATP binding, cell morphogenesis, Golgi organization, cell cycle, signal transduction, cell division, chromosome, centromeric region, spindle, and nucleus
*Vcp*	valosin containing protein	−3.85	1.0 × 10^−4^	protein binding, ATP binding, lipid binding, DNA repair, autophagy, apoptotic process, DNA damage response, nucleus, cytosol, myelin sheath, synapse, and glutamatergic synapse
*Ppp1r1a*	protein phosphatase 1, regulatory (inhibitor) subunit 1A	−3.8	5.02 × 10^−6^	Protein phosphatase inhibitor activity, protein binding, signal transduction, extracellular space, and cytoplasm
*Klf12*	Kruppel-like factor 12	−3.38	6.94 × 10^−7^	DNA binding, regulation of transcription by RNA polymerase II, apoptotic process, protein binding, DNA binding, regulation of transcription by RNA polymerase II, nucleus, and cytosol
*Papola*	poly (A) polymerase alpha	−3.31	3.74 × 10^−5^	nucleotide binding, RNA binding, protein binding, ATP binding, mRNA polyadenylation, and nucleus
*Gng12*	guanine nucleotide binding protein (G protein), gamma 12	−2.98	6.72 × 10^−5^	G-protein beta-subunit binding, signal transduction, actin filament, and membrane
Condition 4				
*Peg3*	paternally expressed 3	−4.79	3.88 × 10^−7^	DNA-binding transcription repressor activity, RNA polymerase II-specific, apoptotic process, nucleus, cytoplasm, and autophagosome
*Vcp*	valosin containing protein	−3.69	1.86 × 10^−7^	protein binding, ATP binding, lipid binding, DNA repair, autophagy, apoptotic process, DNA damage response, nucleus, cytosol, myelin sheath, synapse, and glutamatergic synapse
*Papola*	poly (A) polymerase alpha	−3.49	4.62 × 10^−6^	nucleotide binding, RNA binding, protein binding, ATP binding, mRNA processing, and nucleus
*Rassf3*	Ras association (RalGDS/AF-6) domain family member 3	−3.48	9.25 × 10^−6^	signal transduction, cytoplasm, cytosol, and cytoskeleton
*Erdr1*	erythroid differentiation regulator 1	−3.31	4.32 × 10^−5^	negative regulation of cell population proliferation and negative regulation of cell migration
*Ppp1r1a*	protein phosphatase 1, regulatory (inhibitor) subunit 1A	−3.11	1.29 × 10^−5^	protein phosphatase inhibitor activity, protein binding, carbohydrate metabolic process, signal transduction, extracellular space, and cytoplasm
*Klf12*	Kruppel-like factor 12	−2.98	2.85 × 10^−6^	DNA binding, protein binding, nucleus, and cytosol
*Gpr158*	G protein-coupled receptor 158	−2.97	1.0 × 10^−6^	transmembrane signaling receptor activity, positive regulation of neurotransmitter secretion, signal transduction, brain development, regulation of synapse organization, cognition, and postsynaptic membrane
*Gng12*	guanine nucleotide binding protein (G protein), gamma 12	−2.9	3.44 × 10^−5^	G-protein beta-subunit binding, signal transduction, actin filament, and membrane
*Nedd4l*	neural precursor cell expressed, developmentally down-regulated gene 4-like	−2.84	5.62 × 10^−6^	ubiquitin-protein transferase activity, protein binding, transferase activity, ubiquitin-dependent protein catabolic process, protein ubiquitination, cell differentiation, cytoplasm, and Golgi apparatus

## Data Availability

All data generated or analyzed during this study are included in this published article and its Supplementary Information Files. Microarray data are deposited in the Gene Expression Omnibus (GEO) under Accession Number: GSE236414 (https://www.ncbi.nlm.nih.gov/geo/query/acc.cgi?acc=GSE236414) (accessed on 4 July 2023).

## Supplementary Materials

The following supporting information can be downloaded at: https://www.mdpi.com/article/10.3390/ijms241813867/s1.
