# Supplementary material for: Investigation into Molecular Brain Aging in Senescence-Accelerated Mouse (SAM) Model Employing Whole Transcriptomic Analysis in Search of Potential Molecular Targets for Therapeutic Interventions
Source: Int J Mol Sci. 2023 Sep 8;24(18):13867. doi: 10.3390/ijms241813867 (PMC10530366; doi:10.3390/ijms241813867)
Supplement: Supplementary file 1 [file ijms-24-13867-s001.zip › Supplementary Figure S1.pdf]

# A

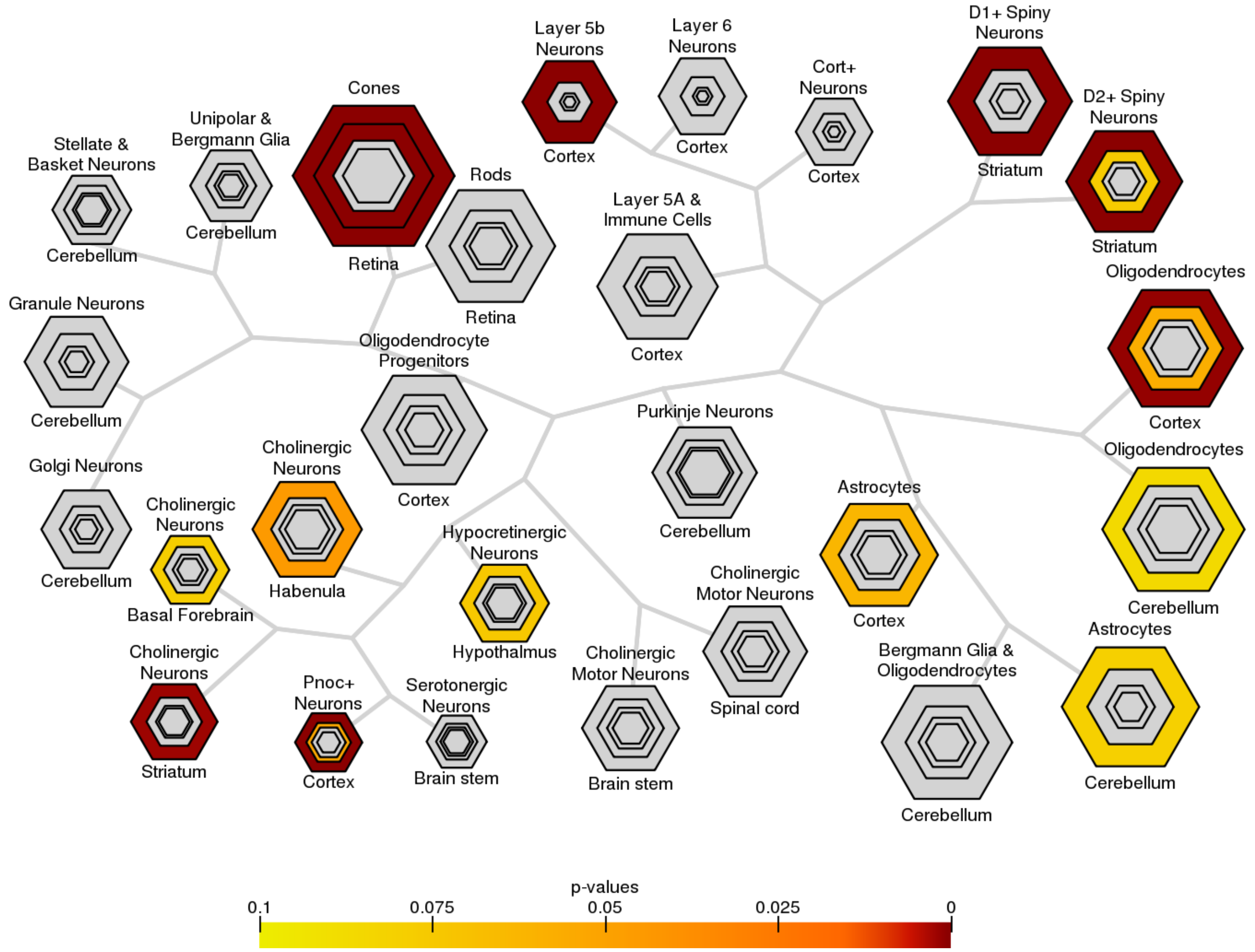


**Supplementary Figure S1: (A)** Brain-related cell-specific enrichment analysis in Condition 1

B

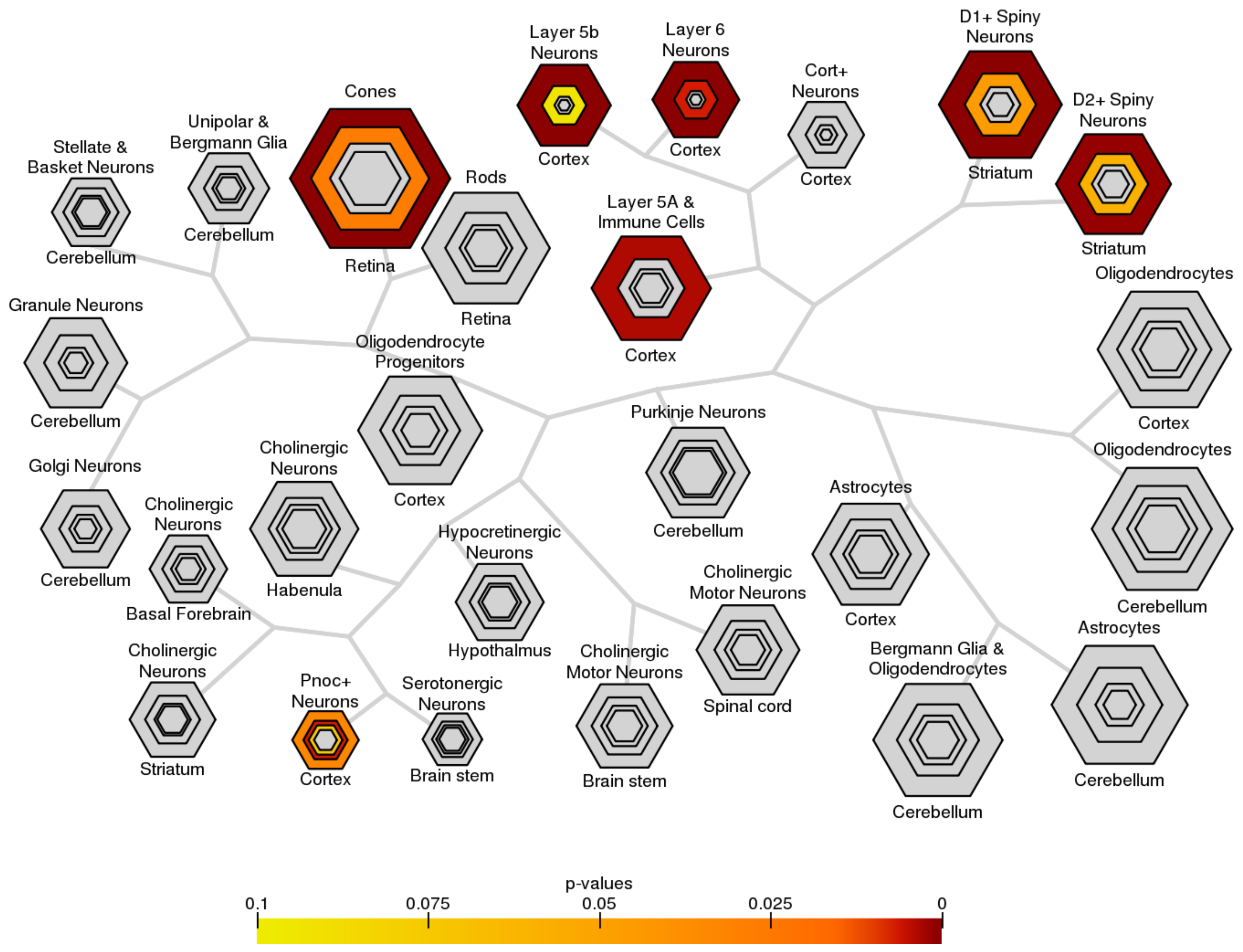


**Supplementary Figure S1:** **(B)** Brain-related cell-specific enrichment analysis in Condition 2

C

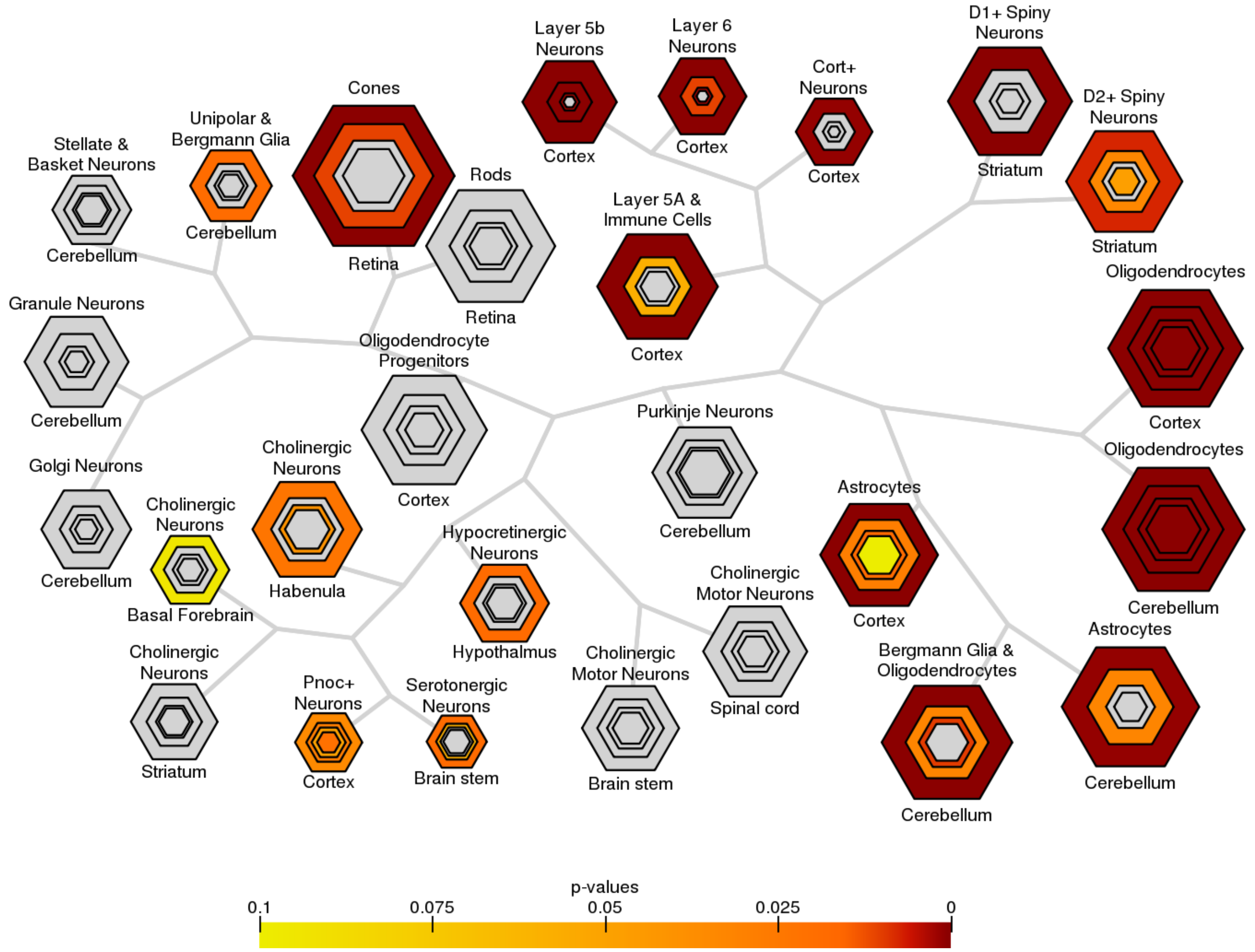


**Supplementary Figure S1: (C)** Brain-related cell-specific enrichment analysis in Condition 3

D

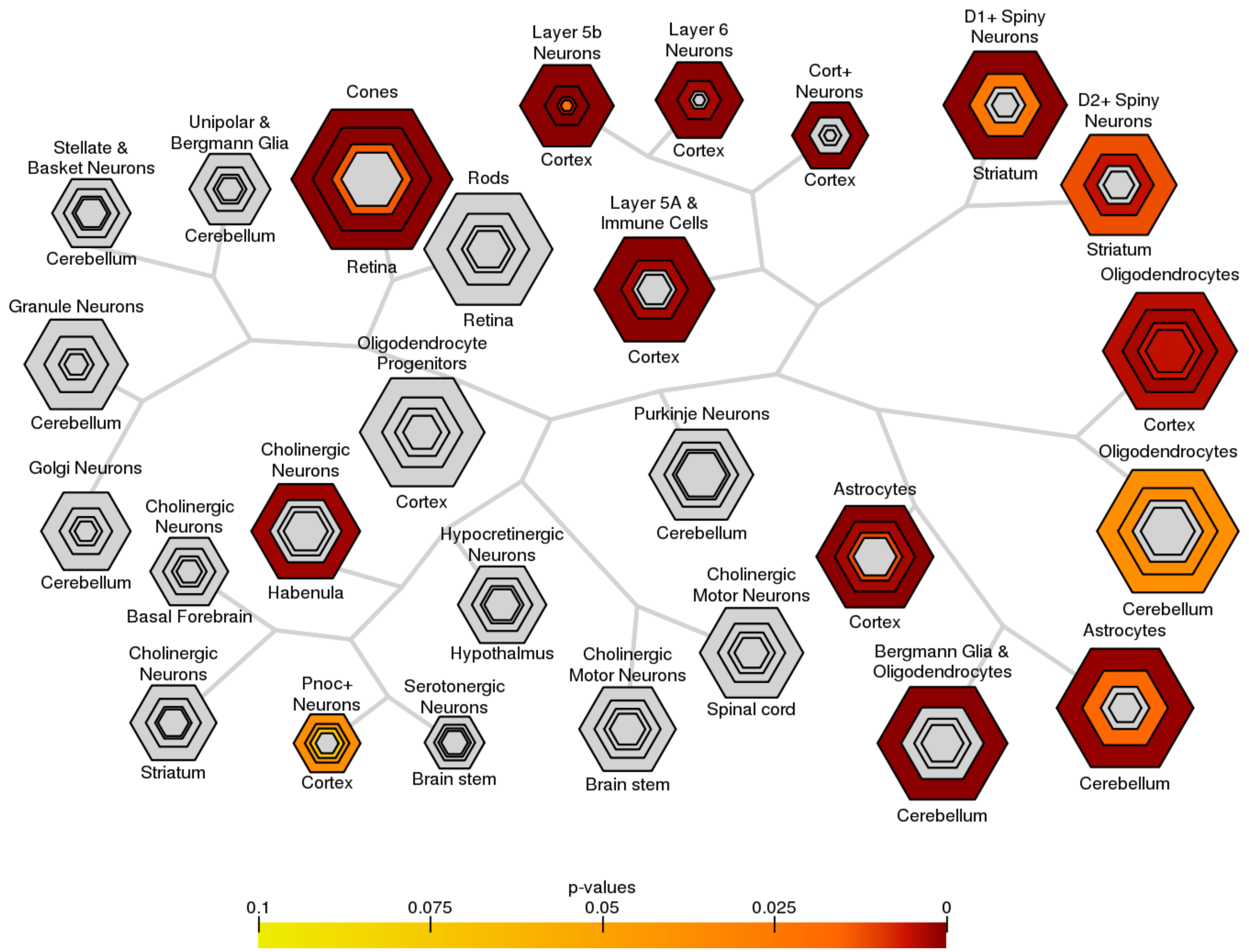


**Supplementary Figure S1: (D)** Brain-related cell-specific enrichment analysis in Condition 4
